# Ablation of TRPC3 disrupts Ca^2+^ signaling in salivary ductal cells and promotes sialolithiasis

**DOI:** 10.1038/s41598-023-32602-8

**Published:** 2023-04-08

**Authors:** Bok-Eum Choi, Samuel Shin, Sade Evans, Brij B. Singh, Bidhan C. Bandyopadhyay

**Affiliations:** 1 Calcium Signaling Laboratory, 151 Research Service, Veterans Affairs Medical Center, 50 Irving Street, NW, Washington, DC 20422 USA; 2 Department of Biomedical Engineering, The Catholic University of America, 620 Michigan Avenue NE, Washington, DC 20064 USA; 3 Department of Periodontics, The University of Texas Health Science Center at San Antonio, 7703 Floyd Curl Dr, San Antonio, TX 78229 USA

**Keywords:** Biochemistry, Cell biology, Physiology, Biomarkers, Diseases, Molecular medicine, Pathogenesis

## Abstract

Clinical studies and structural analyses of salivary stones strongly suggest a linkage between higher saliva calcium (Ca^2+^) and salivary stone formation, *sialolithiasis*; however, the process and the mechanism leading to Ca^2+^ overload during sialolithiasis is not well understood. Here, we show that TRPC3 null (−/−) mice presented with a reduction in Ca^2+^ entry and current in ductal cells with higher saliva [Ca^2+^] suggesting diminished transepithelial Ca^2+^ flux across the salivary ductal cells, leaving more Ca^2+^ in ductal fluid. Significantly, we found that TRPC3 was expressed in mice and human salivary ductal cells, while intraductal stones were detected in both mice (TRPC3^−/−^) and patient (sialolithiasis) salivary glands. To identify the mechanism, we found that TRPC3 was crucial in preventing the expression of calcification genes (BMP2/6, Runx2) in ductal cells which may be due to higher extracellular Ca^2+^ in SMG tissues. Similarly, inflammatory (IL6, NLRP3), fibrotic (FN1, TGFβ1) and apoptotic (Bax1/Bcl2) markers were also elevated, suggesting that the loss of TRPC3 induces genetic changes that leads to salivary gland cell death and induction of inflammatory response. Overall, ablation of TRPC3^−/−^ leads to higher saliva [Ca^2+^], along with elevated detrimental gene expressions, altogether contributing to salivary gland stone formation.

## Introduction

Sialolithiasis is a condition that involves the formation of calcified stone masses (sialoliths) in the salivary gland that eventually obstructs saliva flow and is considered to be one of the most commonly diagnosed salivary gland diseases^1^. A large percentage of these sialoliths are found most frequently in the submandibular gland (SMG) ducts^2^. However, the exact etiology and pathogenesis of calculi formation are mostly unknown. Importantly, shock wave lithotripsy is a common procedure performed without damaging the adjacent tissue^3^. Although, failure of this procedure and the large size of an intraglandular stone often limit treatment and lead to the removal of the gland. Both of these therapeutic options carry risks such as use of anesthetic, facial nerve damage, surgical scar, Frey's syndrome, and dry mouth^4^. Therefore, understanding the molecular and cellular basis of sialolithiasis will help us to pinpoint the etiology and pathogenesis of this disease. It has been proposed that the genesis of Ca^2+^ rich saliva causes relative stagnation of salivary flow. This result in a deposition of Ca^2+^ salts around an initial organic nidus consisting of altered salivary mucins, bacteria, and desquamated epithelial cells, with subsequent progressive accretion forming a stone^5^.

We investigated the calcium phosphate (CaP) stone formation in a clinical setting and found the coexistence of ductal Ca^2+^ stones and vascular calcification in human salivary glands^6^. Furthermore, our studies on kidney stones revealed that the increased [Ca^2+^] in kidney tubular fluid can develop the risk of CaP crystal formation^7^, while elevated tubular epithelial cells [Ca^2+^]_i_ invokes changes in Ca^2+^ signaling pathways and develop detrimental process by activating excessive reactive oxygen species (ROS) production, and ultimately apoptosis^8^. Moreover, both high Ca^2+^ diet or high luminal pH, exhibited a greater increase in Ca^2+^ entry, along with inflammation, oxidative stress, apoptosis, and fibrosis^9,10^. Since calcium overload is followed by an abnormal calcium homeostasis, leading to cell injury and apoptosis, the cell disintegration frequently serves as the nidus of calcification^11^. Importantly, apoptosis could also lead to inflammation, that can also induce calcification along with fibrosis^12^. Apoptosis was evident before the onset of calcification and was followed by an abnormal Ca^2+^ and P_i_ homeostasis to induce cell injury and apoptosis^13^. Calcification can also provoke immune response to injury site where tissue damage triggers an inflammatory response followed by collagen breakdown and calcification^14^. Moreover, calcium-containing crystals can also provoke inflammation by triggering the secretion of inflammatory cytokines^15^.

Experimental and clinical studies strongly suggest a link between high calcium concentration ([Ca^2+^]) in saliva and sialolithiasis. Sialolithiasis starts with the formation of calculi; and patients with calculi and sialolithiasis are shown to have higher [Ca^2+^] in their saliva^16^. We found that SMG ductal cells expressed calcium-sensing receptor (CaSR) which was co-localized with transient receptor potential canonical 3 (TRPC3) ion channel and mediated Ca^2+^ entry at the apical membrane^17^. Such process may constitute the transcellular pathway to represent a potential Ca^2+^ reabsorption mechanism that can account for the decrease in [Ca^2+^] in primary saliva as it flows down the ductal system. Additionally, SMG tissues from sialolithiasis patients revealed a greater area of fibrosis, inflammation, calcification, and apoptosis compared to the normal salivary gland^12^. Studies of intracellular microcalcification in ion-transporting epithelia revealed pathophysiological Ca^2+^ signaling pathways, in which disruption of Ca^2+^ regulation resulted endoplasmic reticulum (ER) and oxidative stress^11,18,19^. However, the link between disruption of Ca^2+^ signaling and the ROS mediated downstream mechanism (ER stress or oxidative stress) in SMG ductal cells is unclear. In this study, we utilized our TRPC3 knock out (KO; ^−/−^) mouse model to examine the physiological implications of the TRPC3 knockdown, and how its compromised Ca^2+^ signaling could potentially lead to fibrosis, inflammation, calcification, and apoptosis within the SMG tissue, while demonstrating an important role that TRPC3 has in potentially mitigating these effects, and eventually preventing stone formation in the salivary gland.

## Materials and methods

### Reagents and chemicals

L-Phenylalanine (L-Phe), Pyr6, and Pyr10 were purchased from Sigma-Aldrich (St Louis, MO). Fura-2-acetoxymethyl ester (Fura-2-AM) was purchased from Invitrogen (Carlsbad, CA). All the chemicals used were analytical grade.

### Animals

Experimental procedures for mice are part of an approved protocol (MIRB-01422) designed according to the Guiding Principles in the Care and Use of Animals and were approved by the Institutional Animal Care and Use Committee (IACUC) and the Research and Development Committee of DC Veterans Affair Medical Center. Both Wild Type (WT) and TRPC3^-/-^ mice were purchased from the Jackson Laboratory (Bar Harbor, ME, USA) and were maintained and crossed previously described^7^. Mice were anesthetized using i.m. injection of ketamine [60–80 mg/kg] and xylazine [8–10 mg/kg] before subcutaneous injection of pilocarpine. All mice were euthanized by CO_2_ asphyxiation from a compressed gas tank followed by cervical dislocation (secondary measure). Animals were euthanized by gradual displacement in home cage to ensure comfort of the animal and no sudden change in environment. The displacement rate for CO_2_ was at a rate of 30% to 70% displacement per minute. This study has been performed under ARRIVE guidelines.

### Electrophysiology

Whole-cell patch clamp recordings were performed using an EPC-10 digitally controlled amplifier and Patchmaster software (HEKA, Lambrecht, Germany) at room temperature as described^7^. Extracellular solution was comprised of NaCl (140 mM), KCl (4 mM), MgCl_2_ (1 mM), CaCl_2_ (2 mM), D-glucose (5 mM), and HEPES (10 mM; NaOH, pH 7.4). Intracellular solution was comprised of (in mM): CsCl (50 mM), NaCl (10 mM), CsF (60 mM), EGTA (20 mM), and HEPES (10 mM; CsOH, pH 7.2). Data was obtained at 5.00 kHz and filtered at 2.873 kHz. Current–voltage (I–V) characteristic curve was obtained every 3 s, by application of voltage ramps (300 ms) from −100 mV to + 100 mV, with a holding potential of −80 mV. Patch clamp has membrane resistance > 500 MΩ.

### Fura-2 loading and measurement of intracellular [Ca^2+^]

Fura-2 [Ca^2+^]_i_ measurements were performed as described^7,17^. After loading with Fura-2-AM, isolated SMG ductal cells were placed on an IX81 motorized inverted microscope equipped with an IX2-UCB control box (Olympus USA, Center Valley, PA). Time-lapse images were captured using IX81 microscope images that were fed into a C9100-02 electron multiplier CCD camera with an AC adaptor A3472-07 (Hamamatsu, Bridgewater, NJ). Lambda-LS xenon arc lamp and 10–2 optical filter changer (Sutter Inst. Novato, CA) were used as an illuminator capable of light output from 340 and 380 nm to a cutoff of 700 nm. Experiments were performed in a microincubator set at 37 °C with a gaseous mixture of 5% CO_2_/95% air. Ratiometric (340/380) measurements of [Ca^2+^]_i_ were recorded using digital microscopy imaging software (SlideBook version 5.0, 3i, Intelligent Imaging Innovations, Denver, CO). Fura-2 ratiometric fluorescence measurements were recorded at an emission peak absorbance at 500 nm wavelength with an excitation peak absorbance alternating between wavelengths of 340 and 380 nm. Time-lapsed measurements were set to 120–500 time points at a 1 s interval. 50–150 cells were individually selected as regions of interest (background fluorescence automatically subtracted prior to 340/380 ratio calculation and graphing). Capture analyses and normalization were performed offline using Slidebook™ software and further quantitated with statistical analysis using Origin 6.1.

### RT-PCR

Total RNA was extracted from mice SMG ductal cells and tissues as described^7^. Cell pellets were lyzed with TRIzol and the precipitated RNA was collected from the aqueous phase after chloroform addition and was then precipitated with 2-propanol followed by 75% ethanol. Precipitated RNA was purified using a DNase I, Application Grade Kit (Sigma-Aldrich, St. Louis, MO, USA), as per the manufacturer’s instructions. Purified RNA concentration was measured using a nanodrop spectrophotometer (ThermoFisher Scientific, Waltham, MA, USA). cDNA was prepared using GoScript™ Reverse Transcription System (Promega, Madison, WI, USA and PCR Master mix was prepared using GoTaq® Green Master Mix (Promega, Madison, WI, USA) as per the manufacturers’ protocol. The primer sequence list used in this study is shown in Table 1. Thermocycling parameters were: the initial denaturation at 95 °C for 3 min; subsequent PCR cycles (× 35) of denaturation at 95 °C for 30 s, annealing at 55 °C for 30 s, and extension at 72 °C for 45 s; and a final elongation at 72 °C for 5 min.

**Table 1:** List of primers.

Primer	Sequence (sense, antisense)	Product size (bp)
mGAPDH	5′-ACTCCACTCACGGCAAATTC-3′ 5′-TCTCCATGGTGGTGAAGACA-3′	171
mTRPC3	5’-TGACTTCCGTTGTGCTCAAATATG-3’ 5’-CCTTCTGAAGCCTTCTCCTTCTGC-3’	317
mNCX1	5′-CCTTGTGCATCTTAGCAATG-3′ 5′-TCTCACTCATCTCCACCAGA-3′	437
mPMCA1	5′- TGGCAAACAACTCAGTTGCATATAGTGG- 3′ 5’-TCCTGTTCAATTCGACTCTGCAAGCCTCG- 3′	562
mPMCA2	5′-AGATCCACGGCGAGCGCAAT-3′ 5’-CGAGTTCTGCTTGAGCGCGG-3′	557
mPMCA3	5′-AGCTCAAGTGCCTGAAGGAAG-3′ 5’-CTGAAGAGGTAGCTGACTTGG-3′	513
mPMCA4	5’-AAGAAGATGATGAAGGACAACAAC-3’ 5’-GTTGCGTACCATATTGTCTCGGTC-3’	564
mCaBp9	5′-GATCATAGTGGGTTTCAGG-3′ 5′-ATCGCCATTCTTATCCAG- 3′	326
mCalBp28	5′-TGGCATCGGAAGAGCAGCAG-3′ 5′-TGACGGAAGTGGTTACCTGGAAG-3′	210
mCaSR	5′-AAACACCTACGGCACCTGAA-3′ 5′-TTGTAGTACCCAACTTCCTTGAACA-3′	152
mSMa	5′- AGATTGTCCGTGACATCAAGG -3′ 5′- TTGTGTGCTAGAGGCAGAGC-3′	538
mNBCe1	5′-CACTGAAAATGTGGAAGGGAAG-3′ 5′-TTATCACCCTTGTGCTTTGC-3′	544
mCHOP	5′- TCAGATGAAAATGGGGGCACCTA -3′ 5′- TTTCCGCTCGTTCTCCTGCTCCTT -3′	297
mNOX4	5′- CCCAAGTTCCAAGCTCATTTCC-3′ 5′- TGGTGACAGGTTTGTTGCTCCT-3′	112
mFMO2	5′-AGTGGCCTAATCTCTCTGAAG-3′ 5′-CATCGGGAAGTCACTGAAACAG-3′	186
mSCD1	5’- TTCTTGCGATACACTCTGGTGC-3’ 5’- CGGGATTGAATGTTCTTGTCGT-3’	98
mBMP2	5′-TGGAAGTGGCCCATTTAGAG-3′ 5′-TGACGCTTTTCTCGTTTGTG-3′	166
mBMP6	5′- CCCGCCCGGAGTAGTTTTAGC-3′ 5′- AGTGCCCTTCTCCCCTCCATT-3′	168
mOCL	5′- CTGACAAAGCCTTCATGTCCAA-3′ 5′- GCGCCGGAGTCTGTTCACTA-3′	59
mOPN	5′- GATGATGATGACGATGGAGACC-3′ 5′- CGACTGTAGGGACGATTGGAG-3′	148
mRUNX2	5′- CGGCCCTCCCTGAACTCT-3′ 5′- TGCCTGCCTGGGATCTGTA-3′	75
mTGFb1	5′- CTGAGTGGCTGTCTTTTG-3′ 5′- TTGCTGTACTGTGTGTCC-3′	288
mFN1	5′- TGCACGATGATATGGAGAGC-3′ 5′- TGGGTGTCACCTGACTGAAC-3′	93
mNLRP3	5′-AGAGCCTACAGTTGGGTGAAATG-3′ 5′-CCACGCCTACCAGGAAATCTC-3′	116
mIL-1β	5′- TCCATGAGCTTTGTACAAGGA-3′ 5′- AGCCCATACTTTAGGAAGACA-3′	343
mIL-6	5′-TGGAGTCACAGAAGGAGTGGCTAAG-3′ 5′-TCTGACCACAGTGAGGAATGTCCAC-3′	155
mMCP1	5′- AGAGAGCCAGACGGGAGGAA-3′ 5′- GTCACACTGGTCACTCCTAC-3′	520
mNF-κβ	5′- GTGGAGGCATGTTCGGTAGT-3′ 5′- AGCTGCAGAGCCTTCTCAAG-3′	367
mGPX3	5′-TGGCTTGGTCATTCTGGGC-3′ 5′-CCCACCTGGTCGAACATACTT-3′	103
mGPX6	5’-GCCCAGAAGTTGTGGGGTTC-3’ 5’-TCCATACTCATAGACGGTGCC-3’	129
m18S	5′-ACGGAAGGGCACCACCAGGA-3′ 5′-CACCACCACCCACGGAATCG-3′	127
mBax1	5′- GAGACACCTGAGCTGACCTT-3′ 5′- GCACCAGTTTGCTAGCAAAG-3′	244
mBCL2	5′- CTCGTCGCTACCGTCGTGACTTCG -3′ 5′- CAGATGCCGGTTCAGGTACTCAGTC -3′	242
mCasp3	5′- GAGCACTGGAATGTCATCTCGC-3′ 5′- AAGCATACAGGCAGTCAGCCTCC -3′	419
mCasp12	5′- GCTGGCCACATTGCCAATTCCC-3′ 5′- GCCAGACGTGTTCGTCCCTCC-3′	314
mSLC26a	5’-AGATCTTCCTTGCGTCTGC-3’ 5’-GCCTTTCCACATGGTAGTCTC-3’	149

### Protein and immunoblotting

Proteins from SMG WT and TRPC3 KO mice ductal cells were harvested by adding ice-cold PBS containing 1% (v/v) aprotinin (Sigma-Aldrich, St. Louis, MO), and were immediately solubilized with RIPA buffer, which contain protease inhibitors as previously described^20,21^. Protein concentration was quantified using the BioRad protein assay (Bio Rad Laboratories, Hercules, CA). Proteins were detected by western blotting, using anti-CaSR (1:400 dilution), and anti-TRPC3 (1:500) primary antibodies, the required secondary antibody, and treatment with ECL reagent as described previously^21^.

### Immunofluorescence and confocal imaging cells

Patient SMG sections were prepared as described^17^ after fixation and permeabilization, which were then rehydrated, blocked, and incubated with anti-rabbit (anti-TRPC3 and anti-CaSR; 1:200) or anti-mouse (anti-SMa; 1:100) antibodies overnight at 4 °C. Co-localization experiments used anti-CaSR antibody (1:100 dilution) with anti-SMa antibody^17,20^ with appropriate controls for TRPC3 and CaSR antibodies were used instead of the primary antibodies, which did not result any positive staining. Co-localization was determined between the anti-rabbit and anti-mouse antibodies by probing the sections with different secondary antibodies at 1:100 dilutions [Alexa-488 donkey anti-rabbit or anti-mouse (for anti-rabbit or anti-mouse primary antibodies, respectively) and Alexa-568 donkey anti-rabbit or anti-mouse (for anti-rabbit or anti-mouse antibodies, respectively)] for 30 min. Nuclear counterstaining of human SMG sections was performed according to manufacturer’s instructions by mixing a 4',6-diamidino-2-phenylindole (DAPI; ThermoFisher Scientific, Cleveland, OH) working solution of 100 ng/mL diluted in 1 × PBS. After washing the sections once with 1 × PBS, the working solutions was applied to the sections in the dark for 30 min at room temperature. Sections were visualized with Zeiss 710 confocal microscopes and were further analyzed using Zeiss software (Zen 2010). In our labeling controls (for background subtraction), isotypes were used instead of the primary anti-bodies and then the secondary antibodies (Alexa Fluor 488 and/or 568-conjugated secondary antibody; Thermo Fisher Scientific) were added.

### Histochemistry of SMG tissue sections

Human and mice tissue sections (∼5–7 µm) were prepared from SMG tissues collected from mice or biopsied from patients, which were fixed in a 10% formalin solution for 24 h and then dehydrated in ethanol and embedded in paraffin. Histochemistry was performed on paraffin sections of mice SMG tissues, as described previously^12^. Alizarin Red (AR) pH 4.3 (for CaP crystal identification) stainings were performed as previously described^22^. Von Kossa stainings were performed to detected calcifications as described previously^12^. In situ apoptosis in mice tissue sections ﻿were performed using TACS•XL DAB in situ Apoptosis Detection Kit (Trevigen, Gaithersburg, MD, USA), as per manufacturer’s directions and our previously published method on mice kidney tissue section^9^.

### Mice saliva electrolyte measurements

WT and TRPC3^−/−^ mice were anesthetized and whole saliva was collected after stimulation with pilocarpine (0.5 mg/kg) body weight as described^23^. The osmolality and ion concentrations were measured as described^24^.

### Statistical analysis

Data were quantitated in mean ± SEM and were analyzed using two-tailed *t*-test, followed by a one-way ANOVA test as required, in Origin 6.1 software. Significant difference levels were set at *p* < 0.05 (*) *or* *p* < 0.01 (**). Details of all statistical analysis are mentioned in figures and figure legends.

### Ethical approval

Specimens are formalin fixed paraffin embedded (FFPE) de-identified tissue section (biopsy sample, not individually identifiable to any person) from Georgetown University tissue bank through an exempt Institutional Review Board (IRB) protocol (Protocol Number: 2010-423; PI: Bandyopadhyay). This IRB exemption was also approved by the IRB and the Research and Development committee of the Washington DC VA Medical Center.

### Statement

All methods were performed in accordance with the relevant guidelines and regulations.

## Results

### Salivary Ca^2+^ levels are elevated in TRPC3^−/−^ mice

**Figure 1:**
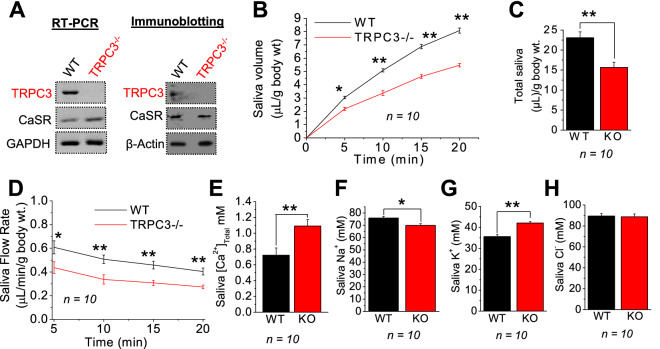
TRPC3 ablation leads to decreased saliva, but increased secretion of Ca^2+^. (**A**) *RT-PCR* and immunoblotting for TRPC3 and CaSR in WT and TRPC3-/- (KO) mice SMG ductal cells were performed with GAPDH and β-actin utilized as internal controls, respectively. Each *RT-PCR* and immunoblotting experiment was performed from *n* = *3* mice. WT and KO mice (*n* = *10)* saliva samples were collected after stimulation with pilocarpine. (**B**, **C**) Volume (total and 0 – 20 min), and (**D**) flow rate (5 – 20 min) were measured and analyzed for WT and KO mice. (**E**) Salivary Ca^2+^, (**F**) Na^+^, (**G**) K^+^, (**H**) Cl^−^ measurements were also obtained from (*n* = *10)* WT and KO mice. Full length blots (**A**) are indicated in the Supplementary Materials. (**C**, **E**–**H**) Bar diagrams are in mean + SEM. *P < 0.05; **P < 0.01.

Abnormal Ca^2+^ homeostasis has been suggested to serve as the nidus of calcification in salivary gland^2,5^. Our previous studies have identified that TRPC3 could assist in renal stone formation; however, the molecular identity of the calcium channel in salivary gland and the mechanism leading to stone formation is not well known. Thus, we evaluated the role of TRPC3 in salivary gland function. Expression of TRPC3 was established at both gene (mRNA levels) and protein levels, where salivary glands isolated from control mice (WT) showed expression of TRPC3, which was absent in TRPC3^−/−^ (Fig. 1A). To further establish the role of TRPC3 in modulating salivary gland function, saliva secretion was evaluated. Parasympathetic stimulation of saliva secretion in mice was initiated using pilocarpine. Importantly, amounts of saliva secretion were consistently diminished in TRPC3^-/-^ mice compared to that of age and gender matched WT mice (Fig. 1B). At the 20 min mark, salivary volume secretion was ~ 35% less in TRPC3^-/-^ mice compared to the WT (5.4 vs. 8.0; Fig. 1B), and by the 20 min mark, total saliva per body weight was also ~ 30% less in TRPC3^−/−^ mice compared to WT mice (15.6 vs. 23.0; Fig. 1C). Moreover, as expected saliva flow rate was also decreased and by the 20 min post stimulation, the rate was further reduced in TRPC3^-/-^ mice by ~ 32% (0.27 vs. 0.40) compared to the WT mice (Fig. 1D). Since saliva secretion was diminished in primary TRPC3^−/−^ mice SMG ductal cells, we performed salivary electrolyte measurements in these mice. Interestingly, while the TRPC3^−/−^ mice had moderately decreased saliva secretion, elevated total Ca^2+^ levels were significantly increased, compared to the WT mice (1.0 vs. 0.72; Fig. 1E). In contrast, other electrolyte levels, such as saliva Na^+^, were decreased in TRPC3^−/−^ as compared to the WT mice (Fig. 1F); while saliva K^+^ levels in the TRPC3^−/−^ mice were slightly increased in TRPC3^−/−^ mice compared to the WT (42.0 vs. 35.5; Fig. 1G). Salivary Cl^−^ levels remained relatively the same between the WT and TRPC3^-/-^ mice (Fig. 1H), suggesting that loss of TRPC3 had elevated Ca^2+^ levels, which could initiate/trigger sialolithiasis^16^.

### CaSR-TRPC3-induced Ca^2+^ entry in SMG ductal cells acts mainly through ROCE

**Figure 2:**
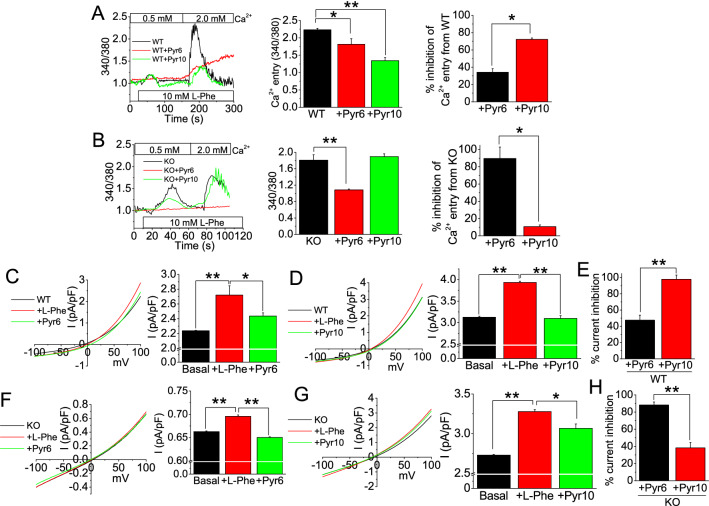
TRPC3 ablation decreases Ca^2+^ entry. Mean ratiometric (340/380 nm) fluorescence [Ca^2+^]_i_ traces were performed on (**A**) WT and (**B**) TRPC3^−/−^ (KO) SMG cells. Prior to experiment, Pyr6 (3 μM) or Pyr10 (3 μM) inhibitor was applied to the cells for 10 min, and the [Ca^2+^]_o_ in the SES buffer was adjusted to 0.5 mM. At start of experiment, for WT cells, L-phenylalanine (L-Phe; 10 mM) was implemented for the Ca^2+^ release at time point ~ 24 s, and then the external solution was adjusted to 2.0 mM Ca^2+^ for the Ca^2+^ entry at time point ~ 155 s for WT cells. For KO cells, L-Phe (10 mM) was implemented for the Ca^2+^ release at time point ~ 11 s, and then the external solution was adjusted to 2.0 mM Ca^2+^ for the Ca^2+^ entry at time point ~ 68 s. Percentage Ca^2+^ entry inhibition comparison of Pyr6 and Pyr10 were performed for (**A**) WT and (**B**) KO mice SMG cells. (**C-E**) WT and (**F–H**) KO SMG cell I-V curves of L-Phe-activated currents with Pyr6 or Pyr10 inhibition, as well as percentage current inhibition by Pyr6 or Pyr10 (**E, H**). Bar diagrams are in mean + SEM. *P < 0.05; **P < 0.01.

While we previously demonstrated that salivary gland ductal cells could elicit Ca^2+^ entry through a CaSR-TRPC3 pathway^17^, the exact mechanism is still unclear. Therefore, to further characterize the CaSR-TRPC3-mediated Ca^2+^ signaling in primary SMG ductal cells, we performed ratiometric (340/380 nm) [Ca^2+^]_i_ measurements, by utilizing L-Phe as a positive allosteric CaSR modulator, and two known pyrazole compounds Pyr6 and Pyr10, to inhibit the store-operated Ca^2+^ entry (SOCE) and receptor operated Ca^2+^ entry pathway (ROCE), respectively^25^. In WT cells, CaSR activation by L-Phe has yielded a small Ca^2+^ release (1.0 to 1.2), followed by a transient Ca^2+^ entry peak (1.0 to 2.23) upon Ca^2+^ replenishment (Fig. 2A). Comparison of Ca^2+^ entries after Pyr6 or Pyr10 incubation revealed greater Ca^2+^ entry diminishment by Pyr10 (from 2.2 to 1.34; 72%) than by Pyr6 (2.23 to 1.81; 34%), suggesting that the majority of the Ca^2+^ entry pathway and possible means of extracellular Ca^2+^ regulation, upon CaSR activation, is through the ROCE pathway (Fig. 2A). In untreated TRPC3^-/-^ ductal cells, CaSR activation yielded a higher Ca^2+^ release than in control WT cells (1.5 vs. 1.2), but also a lower Ca^2+^ entry (1.89 vs. 2.23; Fig. 2B). Preincubation with Pyr10 did not change the Ca^2+^ entry response in KO (TRPC3^−/−^) cells, confirming the absence of TRPC3 (Fig. 2B). In contrast, Pyr6 implementation almost completely blocked the entry (1.8 to 1.0; 89%) responses compared to Pyr10 (1.8 vs. 1.8; 6%) (Fig. 2B), suggesting that the majority of Ca^2+^ entry response is through the SOCE. To confirm our Ca^2+^ imaging findings, whole-cell patch clamp was performed on WT and KO cells (Fig. 2C–H). Patched WT cells revealed an outwardly rectifying current at −80 mV, which is characteristic of a TRPC3 activated current^26^. In WT cells, the L-Phe activated peak current was only partially blocked by Pyr6, while the L-Phe activated current was completely blocked by Pyr10, corroborating with our Ca^2+^ imaging data (Fig. 2A, C–E). On the other hand, in KO cells, the L-Phe activated current was completely blocked by the Pyr6 and partially blocked by Pyr10 (Fig. 2F–H). Overall, these results suggest that SMG ductal cells implements the ROCE within CaSR-TRPC3 pathway as its major transcellular Ca^2+^ regulatory mechanism, while implementing SOCE as its compensatory mechanism, or in this case, after the knockdown of TRPC3.

### TRPC3 inhibits stone formation and calcification in SMG tissue

**Figure 3:**
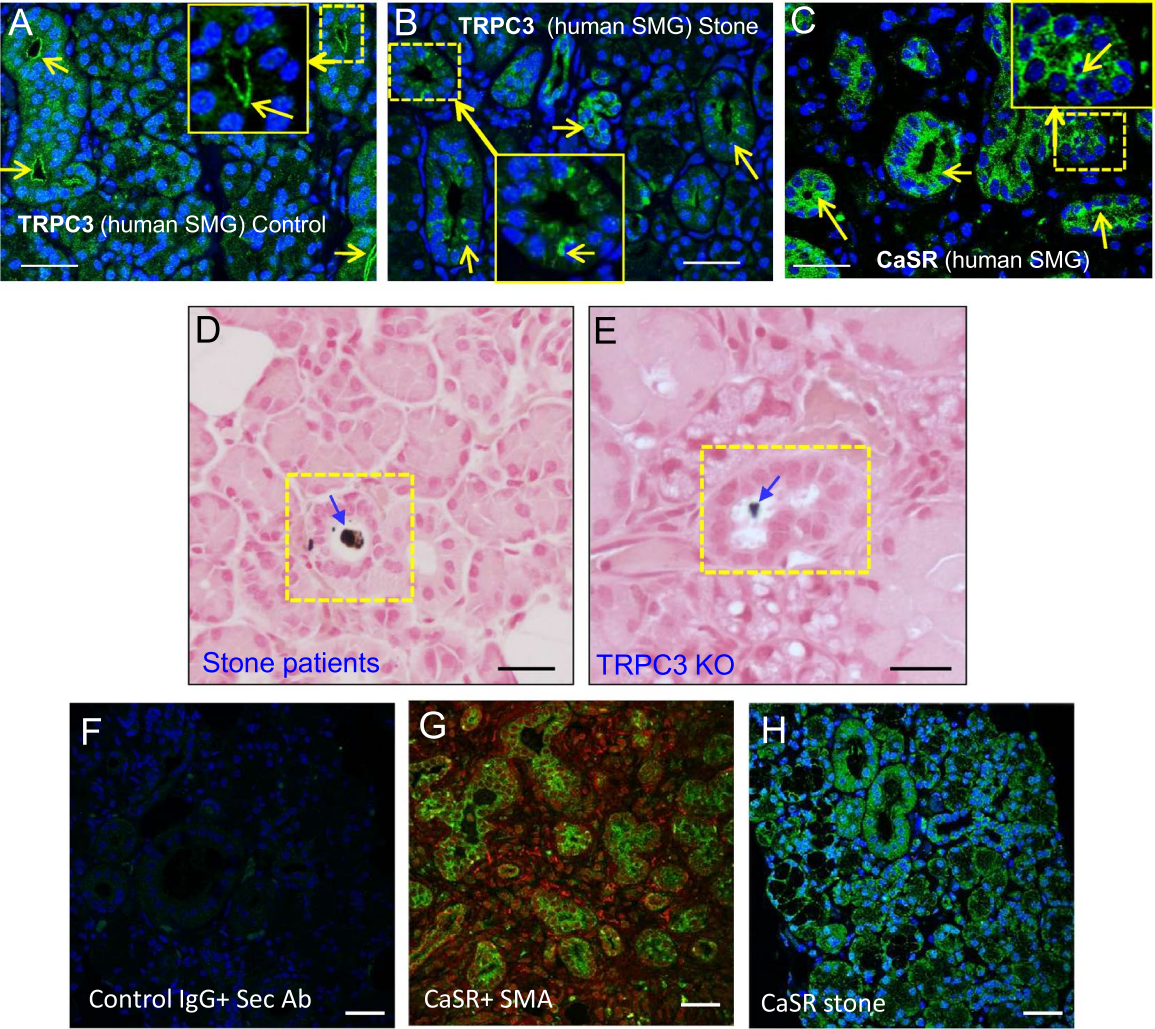
Stone-forming phenotype in TRPC3-ablated mice compared to stone patients. Immunofluorescent images of human (**A**) control and (**B, C**) salivary gland stone patient SMG tissues. TRPC3 or CaSR localization (Green) was depicted with a yellow arrow. Von Kossa staining images of SMG tissue from (**D**) salivary gland stone patient and (**E**) TRPC3 KO mice were acquired with noted calcified areas. (**D**, **E**) Calcified region was indicated with a blue arrow. Immunofluorescent images of SMG tissues portraying (**F**) Control tissue Immunoglobin G (IgG) and secondary antibody (Sec Ab), (**G**) Control CaSR with SMa counterstaining, and (**H**) salivary gland stone patient CaSR. Scale bars = 50 µm (**A-E**) or 35 µm (**F-H**).

Data presented thus far shows that salivary gland ductal cells could regulate extracellular Ca^2+^ through a CaSR-TRPC3 pathway, which may be crucial for regulating Ca^2+^ in salivary ductal fluid thereby limiting stone formation^17^. To further establish the role of TRPC3 in sialolithiasis, we compared the presence of CaSR and TRPC3 in human (non-stone) control and stone patient SMG tissue sections (Fig. 3A–C). TRPC3 appeared to be localized near the apical region of duct epithelial cells in control SMG tissue (Fig. 3A). However, in SMG tissues of salivary stone formers, TRPC3 seemed to be unevenly localized and sparse at the ductal region and were sometimes scattered in areas of the acinus (Fig. 3B). CaSR presence was also confirmed near the ductal region of the SMG (Fig. 3C), indicating that a CaSR-TRPC3 complex could be formed near the ductal site. Since this complex may have a critical involvement in regulating salivary Ca^2+^, we performed Von Kossa staining and compared the region to an analogous TRPC3 KO mice SMG tissue sample (Fig. 3D, E). In both instances, prominent calcification was observed at the ductal site of the SMG that the TRPC3 KO mice could be a translational candidate for salivary stone formation (Fig. 3D, E). In addition, CaSR was congregated in clinical patient samples of apical regions at the ductal sites (Fig. 3F–H), corroborating with our other immunofluorescence findings.

### Stone formation and calcification in SMG due to loss of TRPC3

**Figure 4:**
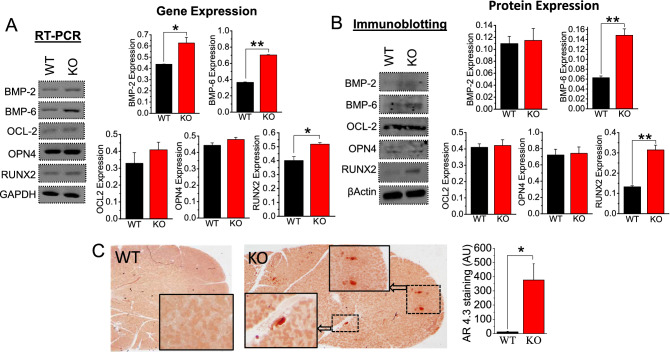
TRPC3-null mice exhibit greater calcification in SMG. Densitometric analysis of (**A**) *RT-PCR* and (**B**) protein blots for calcification markers (BMP-2, BMP-6, OCL2, OPN4, and RUNX2) in WT and TRPC3 KO (KO) mice SMG tissues were performed using ImageJ. GAPDH and β-actin were utilized as internal control for *RT-PCR* and immunoblotting, respectively. Each *RT-PCR* and immunoblotting experiments were performed from *n* = *3* mice. (**C**) WT and KO SMG tissue sections were stained with Alizarin Red (AR; pH = 4.3) to detect calcium crystals. Full length blots are indicated in the Supplementary Materials. (**A–C**) Bar diagrams are in mean + SEM. *P < 0.05; **P < 0.01.

To better understand the mechanism of [Ca^2+^] regulation in saliva, the expression of key transepithelial marker proteins in SMG ductal cells from both WT and TRPC3 KO mice were first examined using RT-PCR. Results revealed that four isomers of plasma membrane calcium ATPase (PMCA), PMCA1, PMCA2, PMCA3, and PMCA4, exist in SMG ducts with no significant difference (*p* > 0.05) in expression levels between WT and KO mice (Fig. S1A). RT-PCR analysis also revealed the presence of Na^+^/Ca^2+^ exchanger isoforms (NCX1) and calbindin (CaBP)-D9k and -D28k as well as anion solute carrier family 26 (SLC26A; Fig. S1B, C) and electrogenic Na^+^/HCO_3_^−^ cotransporter (NBCe1; Fig. S1C). Quantitative results confirmed no compensation sign of gene expression in response to TRPC3 gene knockout, with the exception of the NBCe1 transcript which was elevated (1.3-fold; *p* < 0.05) in SMG ductal cells from TRPC3 KO mice compared to WT mice (Fig. S1C). This observation suggests that increased Na^+^/HCO_3_^-^ cotransport activity in SMG ductal cells may be a contributing factor during pathological conditions caused by the absence of TRPC3^27^. Importantly, Bone morphogenesis (BMP)-2, -6, and -7, osteocalcin 2 (OCL2), osteopontin 4 (OPN4), and runt-related transcription factor 2 (RUNX2) have been known to be osteogenic factors that can lead to stone formation^28,29^. *RT-PCR* analysis showed that the following genes were upregulated in SMG from KO mice when compared to WT mice: BMP2 (0.43 vs. 0.62), BMP6 (0.37 vs. 0.7), and RUNX (0.4 vs. 0.51) (Fig. 4A). However, OCL2 and OPN4 gene expressions were not significantly increased in KO than in WT (Fig. 4A). Similar results were obtained by immunoblot analysis in which protein expression level of BMP-6 in SMG was most significantly upregulated by more than two-fold (2.2-fold; *p* < 0.01) upon absence of TRPC3 (Fig. 4B). TRPC3 knockout had relatively little effect on RUNX2 expression (1.4-fold; *p* < 0.01) and nearly no effect on BMP-2, OCL2, and OPN4 expression in protein expression (Fig. 4B). These finding indicate that BMP-6 is most potent and consistent in inducing osteogenic differentiation and may be involved in mediating terminal osteoblast differentiation of salivary gland cells. Furthermore, comparisons between AR (pH 4.3) stained WT and KO SMG sections revealed diffuse calcified areas in the KO compared to the WT (Fig. 4C), which supports our gene and protein analysis findings (Fig. 4A-C). Together, these findings show that TRPC3 is crucial in preventing calcification due to extracellular Ca^2+^ in SMG tissue.

### Association of salivary gland epithelia cell injury and apoptosis with SMG stone formation

**Figure 5:**
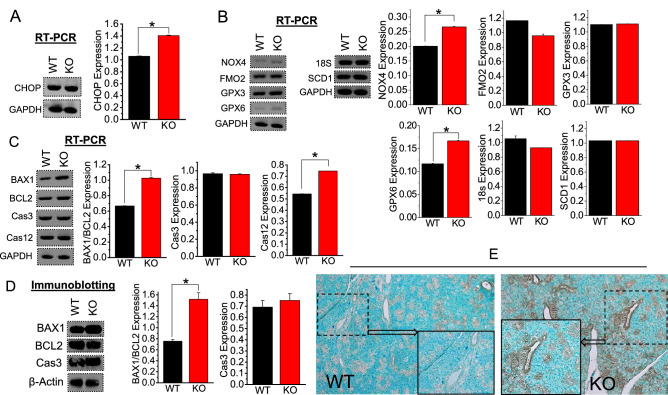
Calcification due to TRPC3 ablation upregulates oxidative stress and apoptosis in SMG. Densitometric analysis of (**A–C**) *RT-PCR* and/or (**D**) protein blot expressions for oxidative stress markers (CHOP, NOX4, FMO2, GPX3, GPX6, m18S, and SCD1) or apoptotic markers (BAX1/BCL2, Cas3, and Cas12) in WT and TRPC3*-/-* (KO) mice SMG tissues were performed using ImageJ. GAPDH and β-actin were utilized as internal control for *RT-PCR* and immunoblotting, respectively. Each *RT-PCR* and immunoblotting experiment was performed from *n* = *3* mice. Full length blots are indicated in the Supplementary Materials. (**A–D**) Bar diagrams of quantitated *RT-PCR* or western blots depicted in mean + SEM. *P < 0.05; **P < 0.01. (**E**) in situ apoptosis was detected in mice WT and KO SMG tissues.

To investigate a link between SMG stone formation and accompanying ROS-induced cell injury and apoptosis, we determined the expression of genes associated with oxidative stress (CHOP, NOX4, FMO2, GPX3, GPX6, m18S, and SCD1; Fig. 5A, B) and apoptosis (B-cell lymphoma protein 2 (BCL-2), BCL-2-associated X (BAX), caspase-3 (Cas3), and -12 (Cas12; Fig. 5C). Quantitative analysis of both mRNA indicated elevated expressions for CHOP, NOX4, and GPX6, confirming that TRPC3 KO SMG exhibits oxidative stress compared to the WT (Fig. 5A, B). Increased BAX/BCL-2 ratio by 1.5 fold in gene and two folds in protein expressions respectively, in TRPC3 KO mice, suggesting cell injury and apoptosis (Fig. 5C, D). However, the mRNA levels of Cas3 were not significantly changed between WT and TRPC3 KO mice, while Cas12 levels were increased significantly (Fig. 5C). This result was further confirmed by immunoblotting (Fig. 5D) experiments which also showed augmented BAX1/BCL-2 levels, but not Cas3. Moreover, these results were corroborated with our *in-situ* staining of WT and TRPC KO mice SMG tissue sections, where TRPC3 KO sections depicted increased areas of peroxidase tagging (apoptosis indication) compared to WT sections (Fig. 5E). Altogether, our results show that increased [Ca^2+^]_i_ in ductal cells and/or ductal saliva Ca^2+^ could contribute to the induction of oxidative stress and subsequent SMG cell death.

### Inflammation and fibrosis in stone-formed SMG

**Figure 6:**
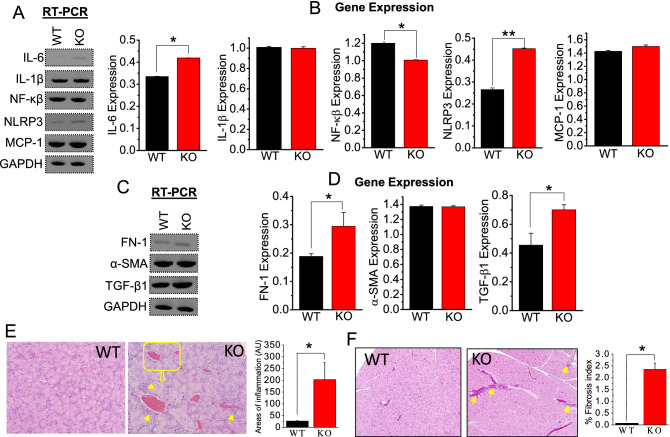
Calcification due to ablation of TRPC3 increases fibrosis and inflammation in SMG. Densitometric analysis of *RT-PCR* expressions for (**A, B**) inflammation markers (IL-6, IL-1β, NF-κβ, NLRP3, and MCP-1) or (**C, D**) fibrotic markers (FN-1, α-SMA, and TGF-β1) in WT and TRPC3^-/-^ (KO) mice SMG tissues were performed using ImageJ. GAPDH was utilized as internal control. Each *RT-PCR* experiment was performed from *n* = *3* mice. (**E, F**) WT and KO SMG tissue sections were stained with H&E to detect inflammation. Full length blots are indicated in the Supplementary Materials. (**A–F**) Bar diagrams of quantitated *RT-PCR*, western blots are depicted in mean + SEM. *P < 0.05; **P < 0.01.

In our previous work, calcified regions of patient SMG tissue were consistent with elevated fibrosis and inflammation^12^, which prompted us to investigate this correlation in our mouse model. To assess an underlying mechanism linking inflammation and fibrosis to calcification and stone formation, semi-quantitative RT-PCR was followed using inflammatory and fibrosis-related gene specific primers. Among the inflammatory genes, the expression levels of NACHT, LRR and PYD domains-containing protein 3 (NLRP3) and interleukin 6 (IL-6) were strongly increased by 1.8 and 1.5-fold in TRPC3 KO mice (Fig. 6A). In contrast, expression of interleukin 1 beta (IL-1β) and monocyte chemoattractant protein-1 (MCP-1) in SMG from TRPC3 KO mice was almost comparable to those from WT mice, and TRPC3 KO gave rise to a slightly decrease in the expression of nuclear factor kappa-light-chain-enhancer of activated B cells (NF-κB; Fig. 6A, B). Our result supports previous findings that the NLRP3 inflammasome can detect and be activated by endogenous danger signals including CaP crystal^11,30^. Next, PCR analysis of fibrosis-associated gene expression revealed that expression of transforming growth factor–β1 (TGF-β1) and fibronectin 1 (FN-1) in SMG ductal cells in TRPC3 KO mice was increase by 1.2-fold while no significant changes were detected for other markers such as α-smooth muscle actin (α-SMA; Fig. 6C, D). TRPC3 KO mice displayed the sign of inflammation as shown in our H&E-stained WT and TRPC3 KO SMG tissue sections (Fig. 6E). Furthermore, Masson's trichrome staining revealed relatively increased collagen deposition associated with a fibrotic response in stroma, which may be secondary to the inflammation (Fig. 6F). Overall, our findings show that fibrosis and inflammation in TRPC3^−/−^ SMG can be linked to inflammatory and fibrotic gene/protein expressions, which can change the overall in SMG microenvironment for playing active role in salivary ductal stone formation^10,27^.

## Discussion

While the biomineralization of CaP is essential to the maintenance of healthy bones and teeth, this process also plays a considerable role in stone formation as the build-up of CaP crystals is the nidus for the development of salivary gland stones^5,6^. In addition, pathological calcifications (e.g., vascular and renal calcifications) which was once believed to be a passive process, is now accepted as an active and highly regulated process similar to bone formation as it shares many of the transcriptional and regulatory signaling pathways^6,31,32^. Salivary gland acinar cells create primary saliva, an isotonic fluid with a relatively high [Ca^2+^] which flows throughout the salivary duct system^33^. Although earlier studies have shown that salivary [Ca^2+^] decreases as it flows down the ductal tree into the oral cavity, ductal reabsorption of Ca^2+^ remains enigmatic^16,34^. Subsequently, the duct modifies the electrolyte composition to produce hypotonic final saliva, called secondary saliva, with no apparent loss in volume^31^. Like in other epithelia, ductal epithelia mediate Ca^2+^ reabsorption via two major pathways, paracellular and transcellular routes^35^. The data presented here clearly shows that loss of TRPC3 not only decreases saliva secretion, but also affect calcium reabsorption, which could be the main factor leading towards stone formation in salivary glands^16,17^. This observation indicates that the overall levels of Ca^2+^ and PO_4_^3−^ in saliva could play in the calcification/stone forming mechanism^7^. Other TRPCs, such as TRPC6 is structurally similar to that of TRPC3 and have been shown to present in apical and basolateral membrane of salivary ductal cells^20,36^. Unlike TRPC3 (which is expressed mainly in the apical membrane), TRPC6 although present apically, it is predominantly basolateral, but TRPC7 did not express in SMG ductal cells^20^. Moreover, we did not see any compensation (increase) in the expression of TRPC6 in TRPC3 KO mice (see Supplemental Material). Moreover, our functional data in Fig. 2 show that TRPC3 is the major conduit in salivary ductal Ca^2+^ reabsorption due to CaSR activation since this activity was severely disrupted in TRPC3 KO mice. Furthermore, Pyr10, the TRPC3 inhibitor, we used, has been found to be more selective to TRPC3 than TRPC6^37^.

Reduction in the amount of saliva secretion as well as elevated Ca^2+^ level in saliva observed in TRPC3^-/-^ mice could also potentially favor the formation of calcium deposits while salivary fluid moving along the duct^17^. Salivary secretion is regulated by the hypothalamus, mainly the lateral hypothalamus (LH) in the central nervous system, which controls SMG secretion through the superior and inferior salivatory nuclei in the brain stem^38^. TRPC channels are highly expressed in hypothalamus to maintain the excitability of hypothalamic neurons^39^. However, it is still unclear whether the absence of TRPC3 in the central nervous system is involved in this process since we collected the saliva after parasympathetic stimulation in global TRPC3^−/−^ mice. It is possible that the systemic application of cholinergic (pilocarpine) agonist that we used to stimulate the parasympathetic secretion in mice may have lost some influence exerted by the LH in global TRPC3 KO mice. Although studies show minimal reduction (~ 20%) in SMG secretion due to lesions in those hypothalamic area, however, it is not clear how much of such reduced in salivation is due to the loss TRPC3 in LH^40^. Moreover, hypothalamic area is highly involved in the mastication-salivary reflex and the involvement of cholinergic pathway or TRPC channel in this process is unknown. Another point is that loss of TRPC3 in acinar cells may be involved in reduced salivation^41^, although drastic reduction in saliva secretion was shown in TRPC1^-/-^mice using similar method^23^. Thus, gradual autonomic denervation in TRPC3^-/-^ mice may be the way to show the influence of salivary center of the midbrain, which is beyond the scope of the present study^38^.

Immunofluorescent staining of mouse SMG sections further revealed distribution and localization of essential components involved in transcellular Ca^2+^ flux and providing evidence that TRPC3 was critical for this process. Interestingly, concomitant increases in Ca^2+^, calcium-binding proteins, P_i_, and phosphatases in blebs form matrix vesicles with membranous cellular degradation products has been shown to result from cell disintegration frequently serves as the nidus of calcification^42^. These previous studies are consistent with our data where increase in apoptosis was observed in TRPC3 KO mice. Moreover, the ER is sensitive to alterations in homeostasis from a variety of different stimuli, including perturbation of calcium homeostasis and CCAAT-enhancer-binding protein homologous protein (CHOP) has been a classical marker for apoptosis^43^. Interesting, increase in CHOP expression, along with increased Bax/Bcl2 ratio was observed in TRPC3 KO mice salivary glands. CHOP, a transcription factor induced under ER stress, has been reported to be involved in ER stress-induced apoptosis by reducing the expression of Bcl-2^44^. Indeed, CHOP-deficiency causes resistance to ER stress-induced cell death both in vitro and in vivo^45^. Caspase-12 is also known to be essential for this ER stress-induced apoptosis, which was also increased in TRPC3 mice^46^. Together, these results suggest that loss of TRPC3 induces salivary gland cell death, which can contribute to stone formation.

We further evaluated as to why cell death was observed. Importantly, previous studies have shown that pathological calcification and chronic inflammation induces an immune response to injury in which tissue damage triggers an inflammatory response followed by collagen breakdown and further calcification driving disease progression^15,47^. Additionally, evidence shows that calcium-containing crystals can provoke inflammation by triggering the secretion of inflammatory cytokines^48^. Published data on physiological calcification are compared with findings in various dystrophic calcinoses. These findings led to the conclusion that apoptosis most likely underlies the mechanism of both physiological and pathological calcifications^42^. Indeed, human SMG stones are shown to be associated with inflammation, fibrosis, and microcalcifications in the surrounding tissues^12^. However, the mechanism underlying tissue degeneration leading to calcification is not fully known. Our data suggest that IL-1β-driven inflammation, that leads to the NLRP3 could be the possible mechanism that is activated upon the loss of TRPC3. Interestingly, crystal induced IL-6 activation was also observed in TRPC3 KO mice kidney cells^9^, suggesting a possible NF-κB–NLRP3–IL-1β pathway. Similarly, elevated IL-6 expression could be supported by our recent findings of ROS-induced activation IL-6 in human kidney cells^48^. Since CaP crystals can trigger the secretion of inflammatory cytokines which themselves are able to trigger the formation of calcifications, it is likely that there is a positive feedback loop between calcification and inflammation^49^.

While SMG calcification and stones co-exists, there is little information about the accompanying cell injury-repair process, apoptosis, and cell proliferation^6^. Fibrosis is primarily driven by inflammatory cytokines including the IL-1β and TGF-b1^50^, which can influence apoptosis^10,12^. Moreover, fibrosis is a normal consequence of tissue injury and chronic inflammation and TGF-β1 is shown to stimulates the NLRP3 inflammasome^51^. Such process is thought to sense the disturbance of cellular homeostasis rather than directly recognizing a common motif present in its activators, and multiple cellular signals have been proposed to trigger its activation, including K^+^ efflux, Ca^2+^ signaling, mitochondrial dysfunction, and lysosomal rupture^11^. Fibrosis and inflammation in lung were also shown to be elicited via a Bax-dependent, Bid-activated Pathway^52^. These results are consistent with our data and taken together, these findings suggest that inflammation was more involved in SMG stone formation. Importantly, IL-6 is a known downstream target of IL-1β and is consistently increased in serum from patients with NLRP3 inflammasome-mediated conditions, elucidating the possible mechanism as how TRPC3 modulates the immune response that may be implied in salivary stone formation.

## Supplementary Information

Supplementary Information.

## Data Availability

All data associated with this study are present in the paper or the Supplementary Materials. The background data that support the findings of this study are available from the corresponding author upon reasonable request.
