# Supplementary material for: Ablation of TRPC3 disrupts Ca2+ signaling in salivary ductal cells and promotes sialolithiasis
Source: Sci Rep. 2023 Apr 8;13:5772. doi: 10.1038/s41598-023-32602-8 (PMC10082769; doi:10.1038/s41598-023-32602-8)
Supplement: Supplementary file 1 — Supplementary Information. [file 41598_2023_32602_MOESM1_ESM.pdf]

SUPPLEMENTAL MATERIALS

Ablation of TRPC3 disrupts Ca^{2+} signaling in salivary ductal cells and promotes sialolithiasis

Bok-Eum Choi[1,§] Samuel Shin[1,2§], Sade Evans[1], Brij B. Singh[3]
and Bidhan C. Bandyopadhyay[1,2]*

[1]Calcium Signaling Laboratory, Research Service, Veterans Affairs Medical Center, 50 Irving Street, NW, Washington DC, 20422. [2]Department of Biomedical Engineering, The Catholic University of America, 620 Michigan Avenue NE, Washington DC, 20064, USA. [3]Department of Periodontics, The University of Texas Health Science Center at San Antonio, 7703 Floyd Curl Dr, San Antonio, TX 78229.

Running Title: *TRPC3 and sialolithiasis*

[§] These authors contributed equally to this study.

*To whom correspondence should be addressed:

Bidhan C. Bandyopadhyay, PhD. Chief, Calcium Signaling Laboratory, 151 Research Service, Veterans Affairs Medical Center, 50 Irving Street, NW, Washington, DC 20422, Phone: (202) 745-8622, Fax: (202) 462-2006; E-mail: bidhan.bandyopadhyay@va.gov

Supplemental Figures

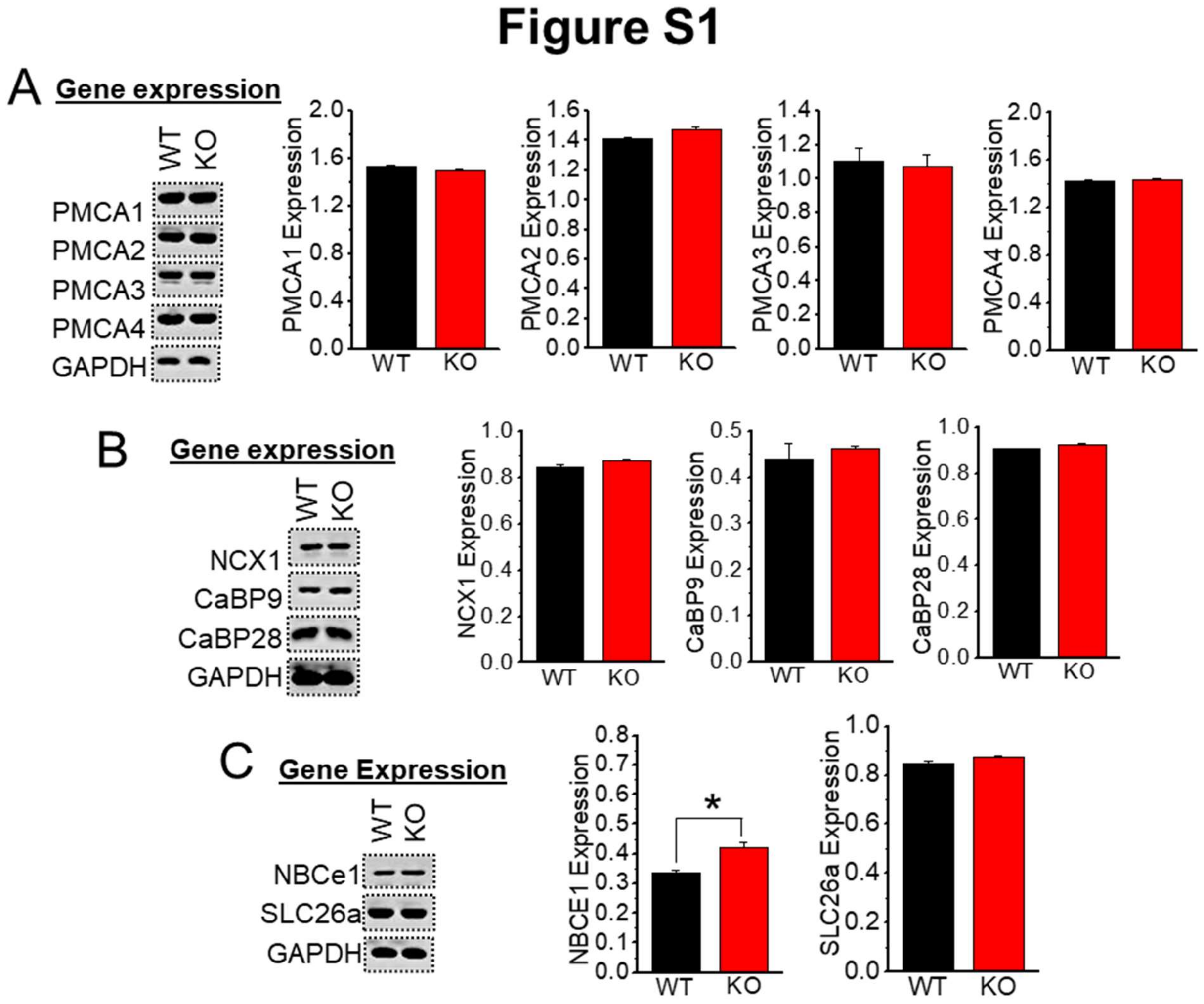

Figure S1. Densitometric analysis of *RT-PCR* expressions for (**A**) Ca^{2+} transporters (PMCA1, PMCA2, PMCA3, PMCA4, NCX1, CaBP9, and CaBP28) or (**B-C**) solute transporters (NBCe1 and SLC26a6) in WT and TRPC3 KO (KO) mice SMG tissues were performed using ImageJ. GAPDH was utilized as internal control. Each *RT-PCR* experiment was performed from *n=3* mice. (**A-C**) Bar diagrams of quantitated RT-PCR, western depicted in mean + SEM. *, P < 0.05; **, P < 0.01.

RT-PCR Blot in Figure 1

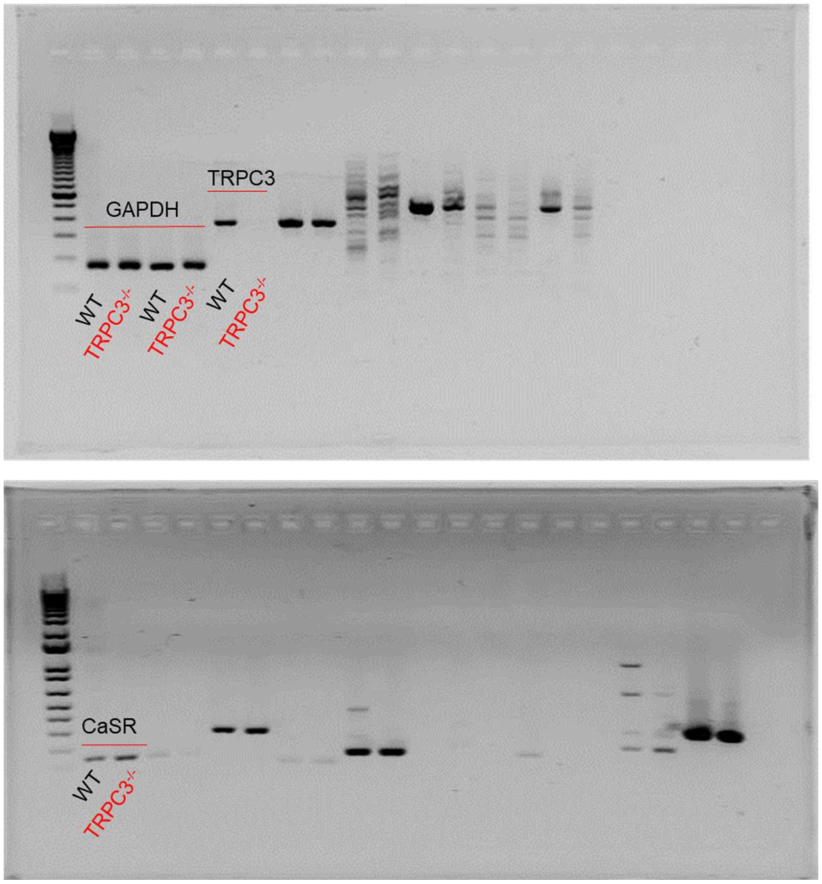

Immunoblotting in Figure 1

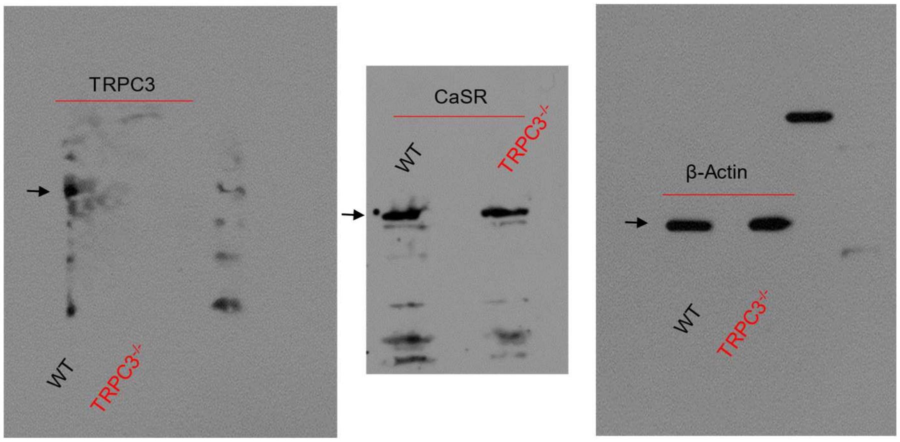

RT-PCR Blot in Figure 4

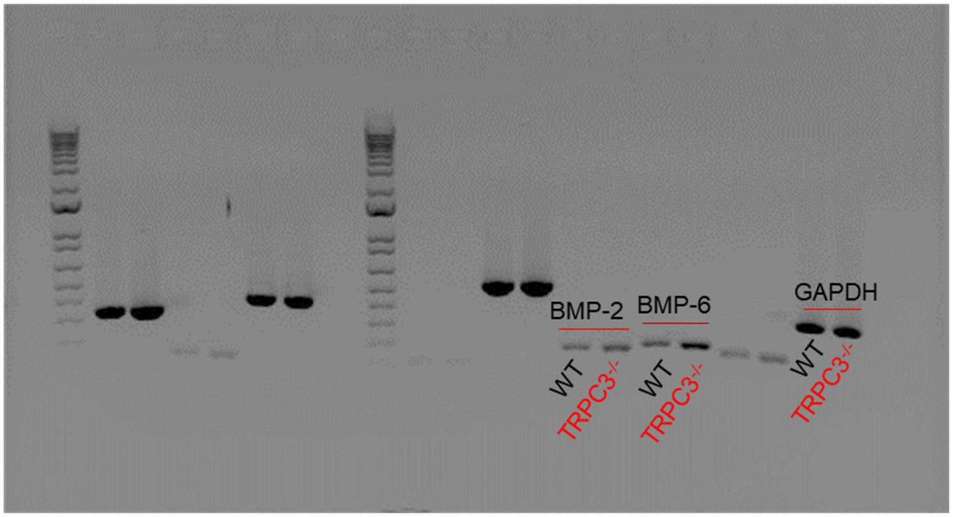

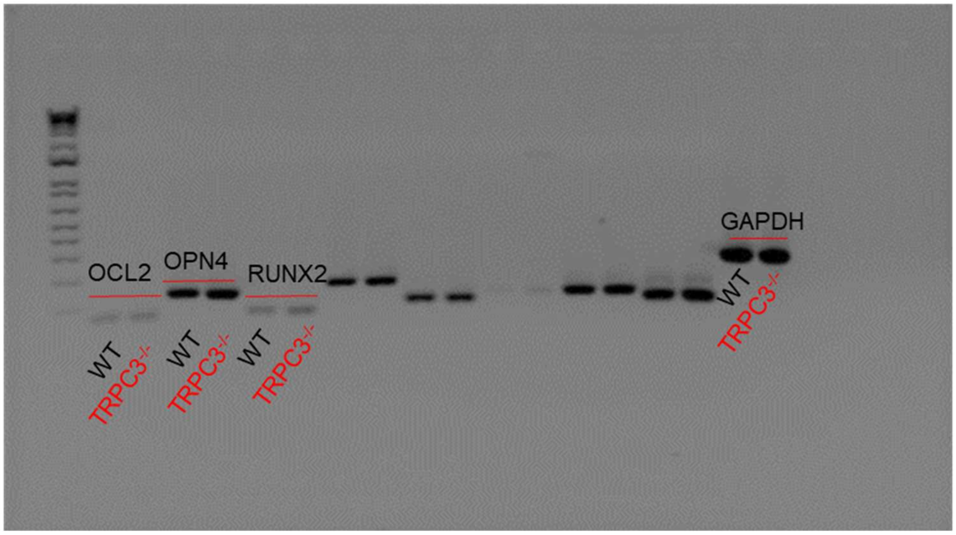

Immunoblotting in Figure 4

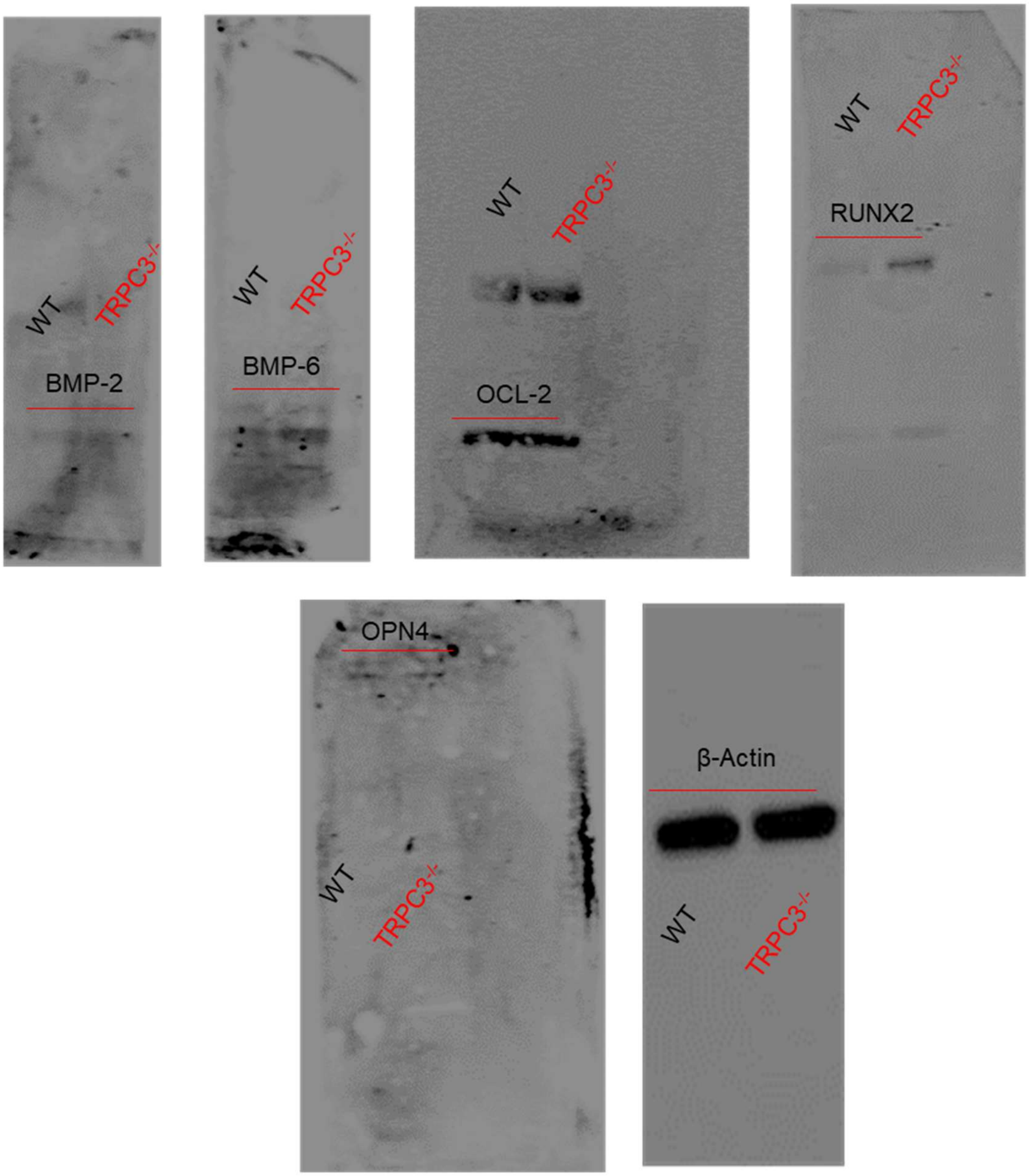

RT-PCR Blot in Figure 5

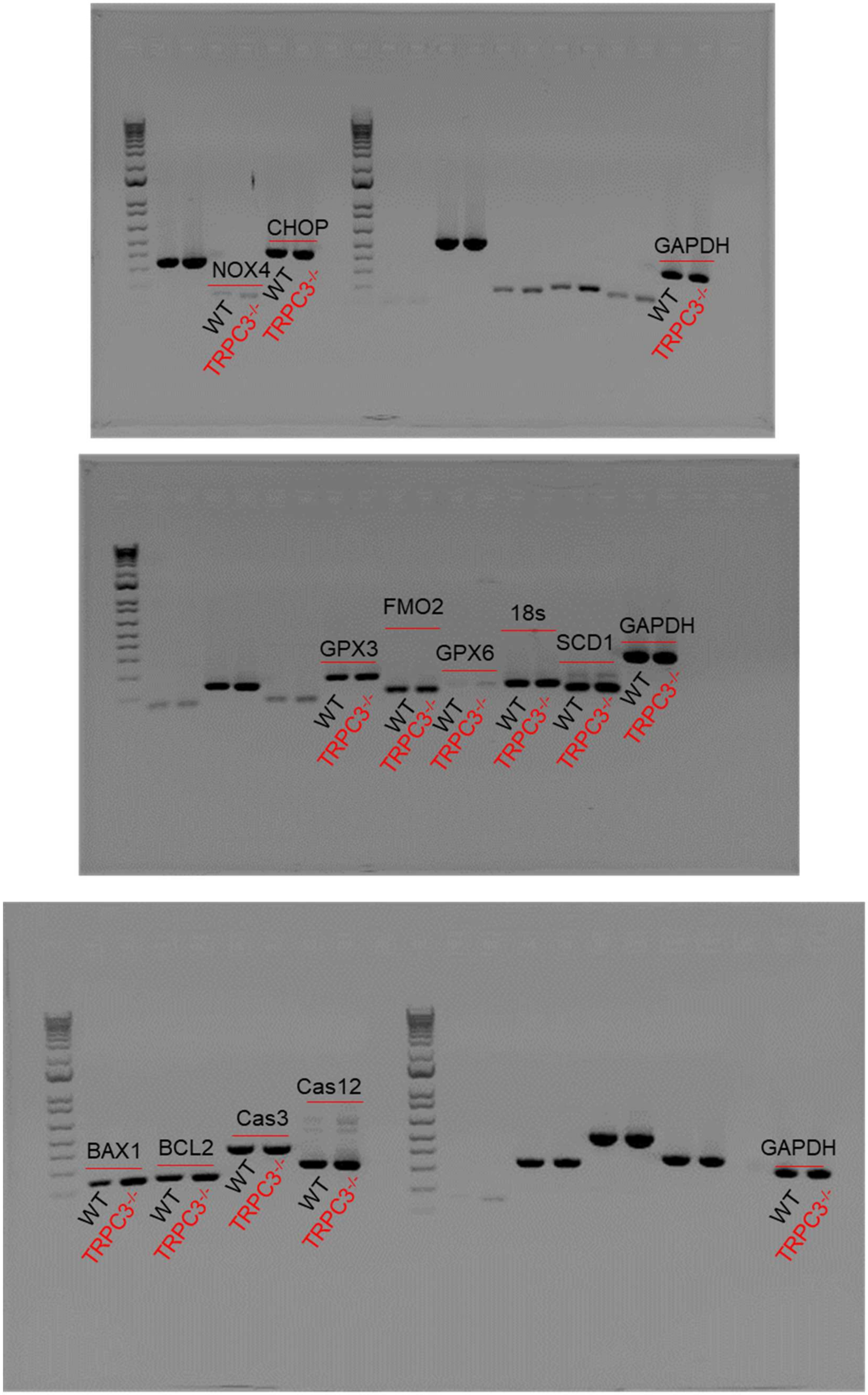

RT-PCR Blot in Figure 5 Cond.

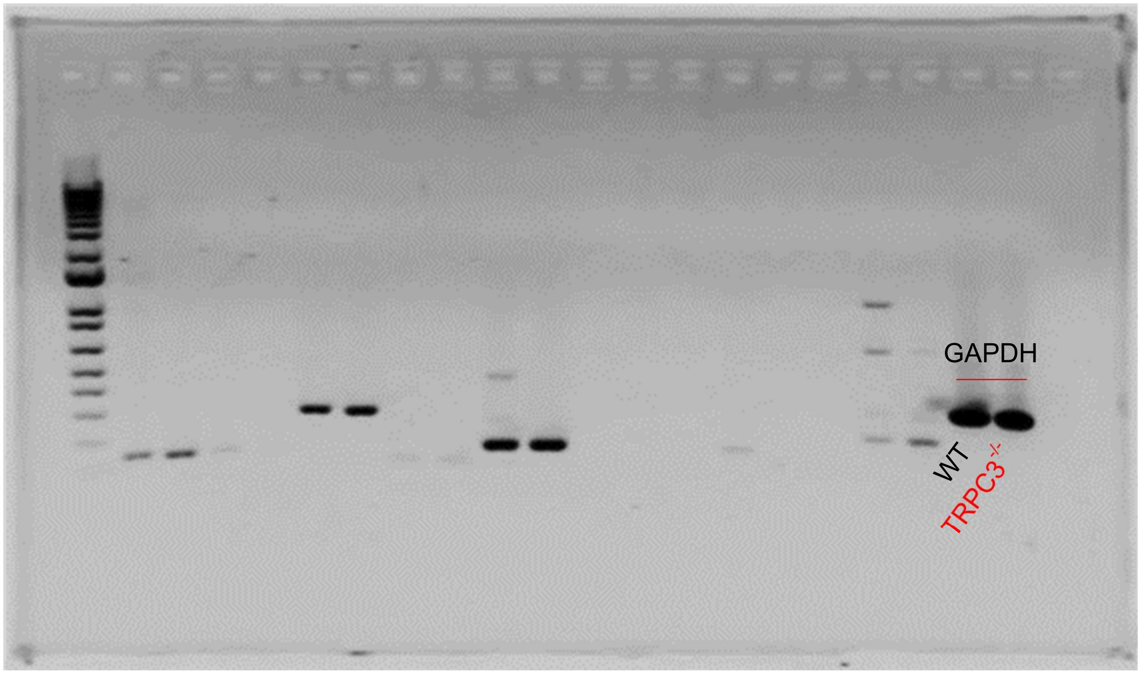

Immunoblotting in Figure 5

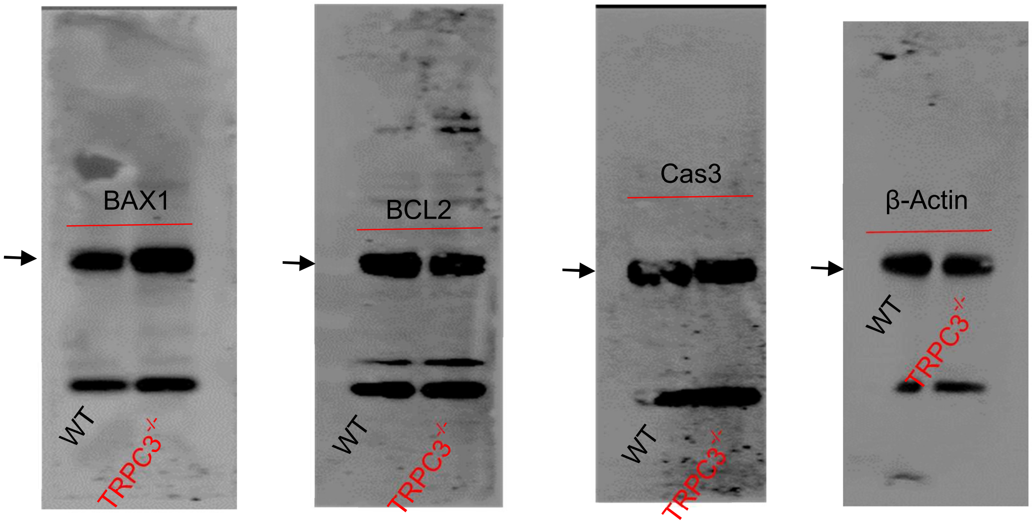

RT-PCR Blot in Figure 6

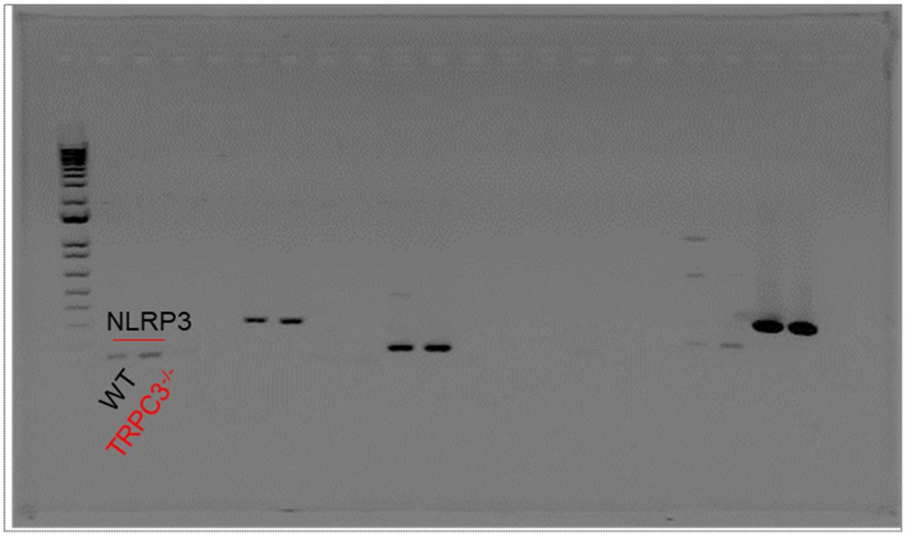

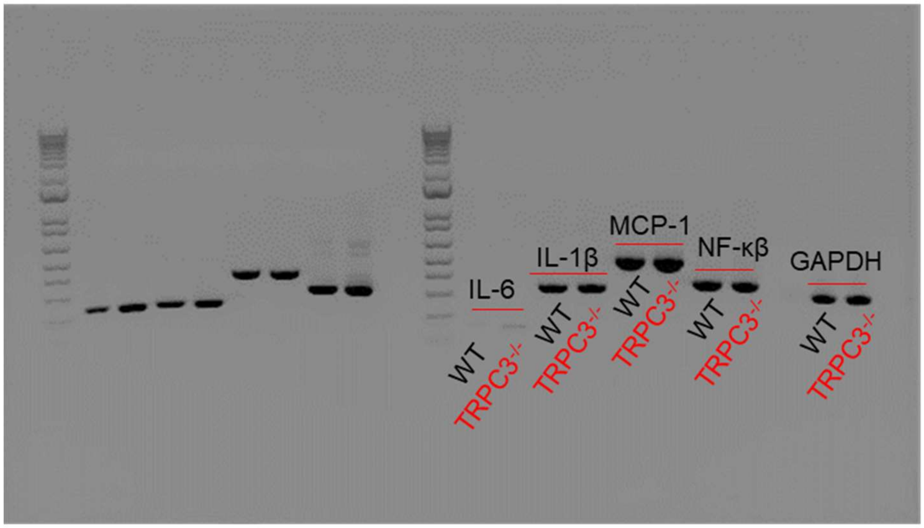

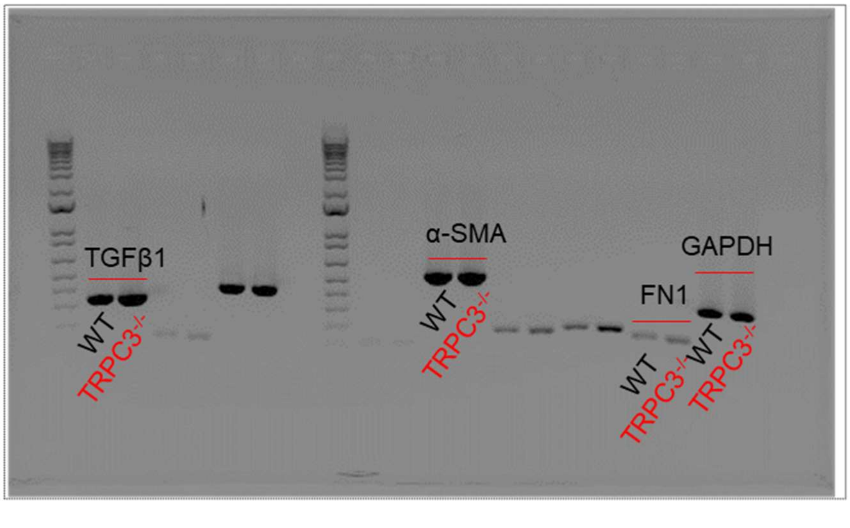

RT-PCR Blot in Figure S1

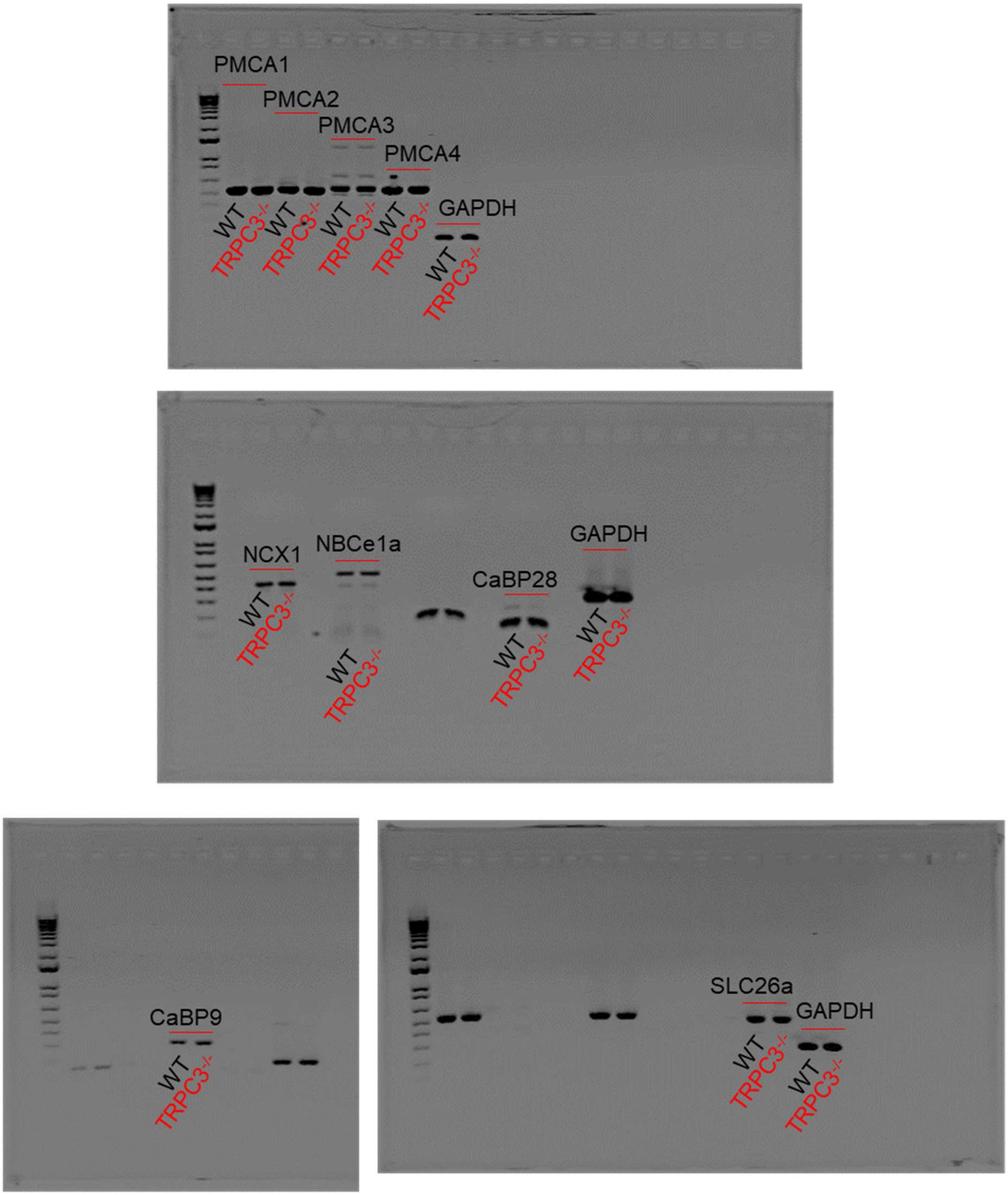

Additional Supplemental material

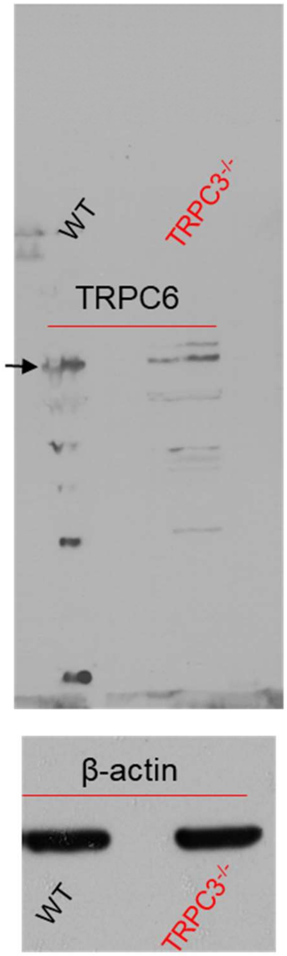

TRPC6 expression in SMG ductal cells: Western blot show the expression of TRPC6 using Anti-TRPC6 antibody (1:500 dilution; Sigma Chemicals, St. Louis, MO) on SMG ductal cell lysate. β-actin was used as a loading control.
